# Use of antiretroviral therapy during pregnancy among HIV-infected women attending an urban care centre

**DOI:** 10.1186/1758-2652-13-S4-P161

**Published:** 2010-11-08

**Authors:** F Ssewankambo, C Nalugwa, J Sempa, J Walusimbi, A Kambugu

**Affiliations:** 1 Infectious Diseases Institute, Prevention Care & Treatment, Kampala, Uganda; 2 Infectious Diseases Institute, Research Department, Kampala, Uganda

## Background

With the large international mobilization of resources against HIV/AIDS and the great improvement of ARV drug access within the last 7 years in resource-limited countries, more HIV-infected people are expected to access antiretroviral therapy (ART) including pregnant women. However, coverage of PMTCT services in many Sub-Saharan countries is still low. The aim of the study was to characterize and estimate the number of pregnant women who were receiving ART at adult Infectious Diseases Institute clinic (AIDC).

## Methods

We retrospectively analyzed routinely collected data in HIV/AIDS clinic within a period of Jan-2008 to Oct 2009 among HIV positive pregnant women attending care at (AIDC). Descriptive data analyses were conducted to characterize and estimate the number of pregnant women on ART at AIDC.

## Summary of results

A total of 649 pregnant women were recorded with median age 30(IQR: 26, 33) years and (196/427) 45% had unintended pregnancy. Ninety-five percent had formal education while 84% were married. Their health status in regard to current median CD4 was 376 (IQR: 282, 550) cells/mm3 while 315 women were on ART (85% NVP-based & 14% PI-based). Among 208 women who had reported to have delivered, only (83/208) 40% knew the sero-status of their babies (79/83) 95% HIV-ve and (4/83) 5% HIV+ve).

## Conclusions

Our results indicate inadequate follow-up of PMTCT service utilization and low documentation of pregnancy outcomes among HIV positive pregnant women attending care. There is a need to strengthen PMTCT and ART integration in already existing HIV/AIDS care program. Establishing linkages with paediatric HIV/AIDS care programs in order to enhance effectiveness of PMTCT services is essential.

